# Postdiagnosis circulating osteoprotegerin and TRAIL concentrations and survival and recurrence after a breast cancer diagnosis: results from the MARIE patient cohort

**DOI:** 10.1186/s13058-023-01625-4

**Published:** 2023-04-17

**Authors:** Charlotte Le Cornet, Audrey Y. Jung, Theron S. Johnson, Sabine Behrens, Nadia Obi, Heiko Becher, Jenny Chang-Claude, Renée T. Fortner

**Affiliations:** 1 Division of Cancer Epidemiology, German Cancer Research Center, Heidelberg, Germany; 2 Cancer Epidemiology Group, University Cancer Center Hamburg, University Medical Center Hamburg-Eppendorf, Hamburg, Germany; 3 Institute of Global Health, University Hospital Heidelberg, Heidelberg, Germany; 4 Department of Research, Cancer Registry of Norway, Oslo, Norway

**Keywords:** OPG, TRAIL, Breast cancer survival, Estrogen receptor status

## Abstract

**Background:**

Experimental studies suggest a role for osteoprotegerin (OPG) and tumor necrosis factor-related apoptosis-inducing ligand (TRAIL) in mammary tumor development and progression. These biomarkers have been minimally investigated with respect to outcomes in breast cancer patients.

**Methods:**

OPG and TRAIL were evaluated in blood samples collected from 2459 breast cancer patients enrolled in the MARIE study, a prospective population-based patient cohort, at median of 129 days after diagnosis. Participants were between ages 50 and 74 at diagnosis and recruited from 2002 to 2005 in two regions of Germany. Follow-up for recurrence and mortality was conducted through June 2015. Delayed-entry Cox proportional hazards regression was used to assess associations between OPG and TRAIL with all-cause and breast cancer-specific mortality, and recurrence, both overall and by tumor hormone receptor status.

**Results:**

Median follow-up time was 11.7 years, with 485 deaths reported (277 breast cancer-specific). Higher OPG concentrations were associated with a higher risk of all-cause mortality (hazard ratio for 1-unit log2-transformed concentration (HR_log2_) = 1.24 (95% confidence interval 1.03–1.49). Associations were observed in women diagnosed with ER-PR- tumors or discordant hormone receptor status (ER-PR-, HR_log2_ = 1.93 (1.20–3.10); discordant ERPR, 1.70 (1.03–2.81)), but not for women with ER + PR + tumors (HR_log2_ = 1.06 (0.83–1.35)). OPG was associated with a higher risk of recurrence among women with ER-PR- disease (HR_log2_ = 2.18 (1.39–3.40)). We observed no associations between OPG and breast cancer-specific survival, or for TRAIL and any outcome.

**Conclusions:**

Higher circulating OPG may be a biomarker of a higher risk of poor outcome among women diagnosed with ER- breast cancer. Further mechanistic studies are warranted.

**Supplementary Information:**

The online version contains supplementary material available at 10.1186/s13058-023-01625-4.

## Introduction

Receptor activator of nuclear factor-κB (RANK) signaling plays a critical role in breast development, mediated by a transmembrane receptor (RANK), its ligand (RANKL) and osteoprotegerin (OPG), a decoy receptor for RANKL (i.e., binds RANKL, keeping RANKL from binding RANK). Experimental research suggests a role for RANK-axis members in breast cancer etiology, treatment, and prevention [1–4]. An earlier study from our group supports an association between OPG and hormone receptor (i.e., estrogen (ER) and progesterone (PR) receptor) negative breast cancer risk [5], though findings from other studies on risk are mixed ([6], reviewed in [7]). In terms of survival, a prior clinical study observed a higher risk of breast cancer-specific death in women with high (above median), relative to low, OPG levels [8], and our prior study evaluating pre-diagnosis OPG concentrations and mortality outcomes reported a higher risk of death with higher OPG [9]. No associations were observed for RANKL, or the RANKL/OPG ratio, in either study.

We hypothesized that the positive association observed between OPG and ER- breast cancer risk in our prior study may be due to the dual role of OPG as the decoy receptor for RANKL and TNF-Related Apoptosis-Inducing Ligand (TRAIL). TRAIL induces apoptosis, particularly in hormone receptor-negative breast cancer cell lines [10–12], and OPG binding to TRAIL decreases TRAIL-induced apoptosis in these cell lines [12, 13]. The interaction between OPG and TRAIL in vivo is unclear [11]. However, higher OPG may increase ER- breast cancer risk and increase the risk of death following diagnosis in part via inhibition of TRAIL-induced apoptosis. Given the sparse data on circulating OPG and TRAIL and survival following a breast cancer diagnosis, we evaluated associations between postdiagnosis circulating OPG and TRAIL concentrations in relation to all-cause mortality, breast cancer-specific mortality, and recurrence-free survival overall and by hormone receptor status in a German population-based patient cohort study.

## Methods

### Study population

This study was conducted in the Mammary Carcinoma Risk Factor Investigation (MARIE) Study, a prospective population-based patient cohort study that began as a case–control study conducted in two regions of Germany: Rhine-Neckar-Karlsruhe and Hamburg [14]. From 2002 to 2005, 3813 patients 50–74 years of age at diagnosis with an incident histologically confirmed invasive breast cancer (ICD-10 C50) (stage I to IV) or in situ tumor (D05) (stage 0) were recruited from participating clinics and cancer registries. To be eligible, patients had to reside in one of the study regions and be capable of participating in a 1.5 h in-person interview. Patients were identified through frequent monitoring of hospital admissions, surgery schedules, and pathology records of all clinics serving these regions and also through the Hamburg Cancer Registry. In 2009, patients were re-interviewed about weight and other exposures and a subset provided a second blood sample, and follow-up information about endpoints was ascertained in 2009 and 2015. The study was approved by the ethics committees of the University of Heidelberg, the State of Rhineland-Palatinate and the Hamburg Medical Council, and was conducted in accordance with the Declaration of Helsinki. All study participants provided informed written consent.

**Fig. 1:**
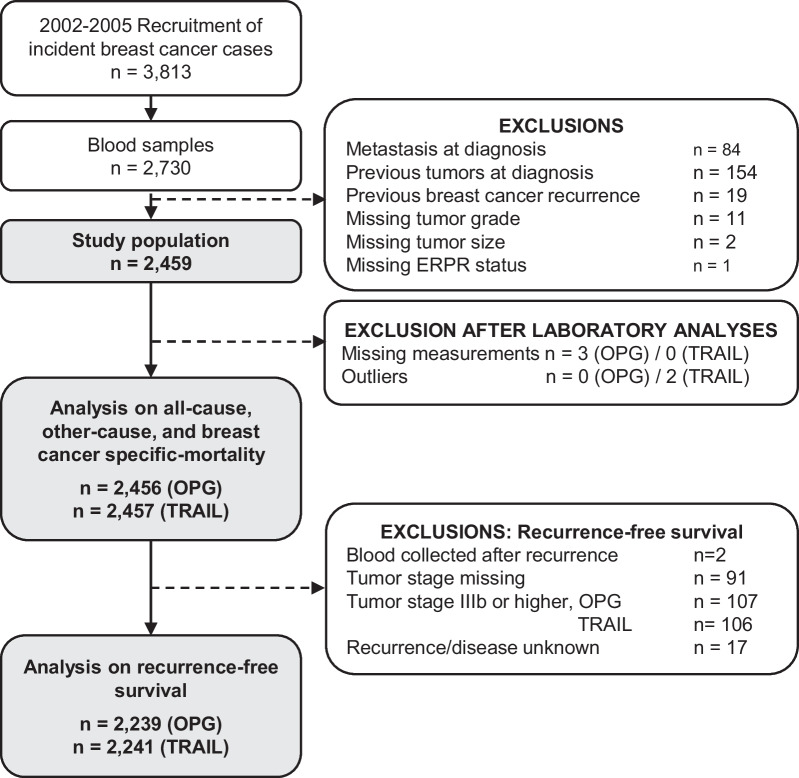
Flowchart of inclusion and exclusion criteria for participants of the MARIE study for analyses relating to OPG and TRAIL concentrations and recurrence and survival

Women who provided blood samples at recruitment and for whom a blood specimen was available (n = 2730) were initially eligible for analyses. Women with metastases at diagnosis (n = 84), previous tumors other than breast cancer or non-melanoma skin cancer before diagnosis (n = 154), previous breast cancer recurrence (n = 19), missing data for tumor grade (n = 11), size (n = 2), and ERPR status (n = 1) were excluded. Additionally, missing measurements (OPG n = 3/TRAIL n = 0) and outliers (OPG n = 0/TRAIL n = 2) were excluded. Overall, 2459 women were available for analysis including 2456 observations with OPG measurements and 2457 with TRAIL (Fig. 1).

### Exposure assessment: biomarker measurements

**Fig. 2:**
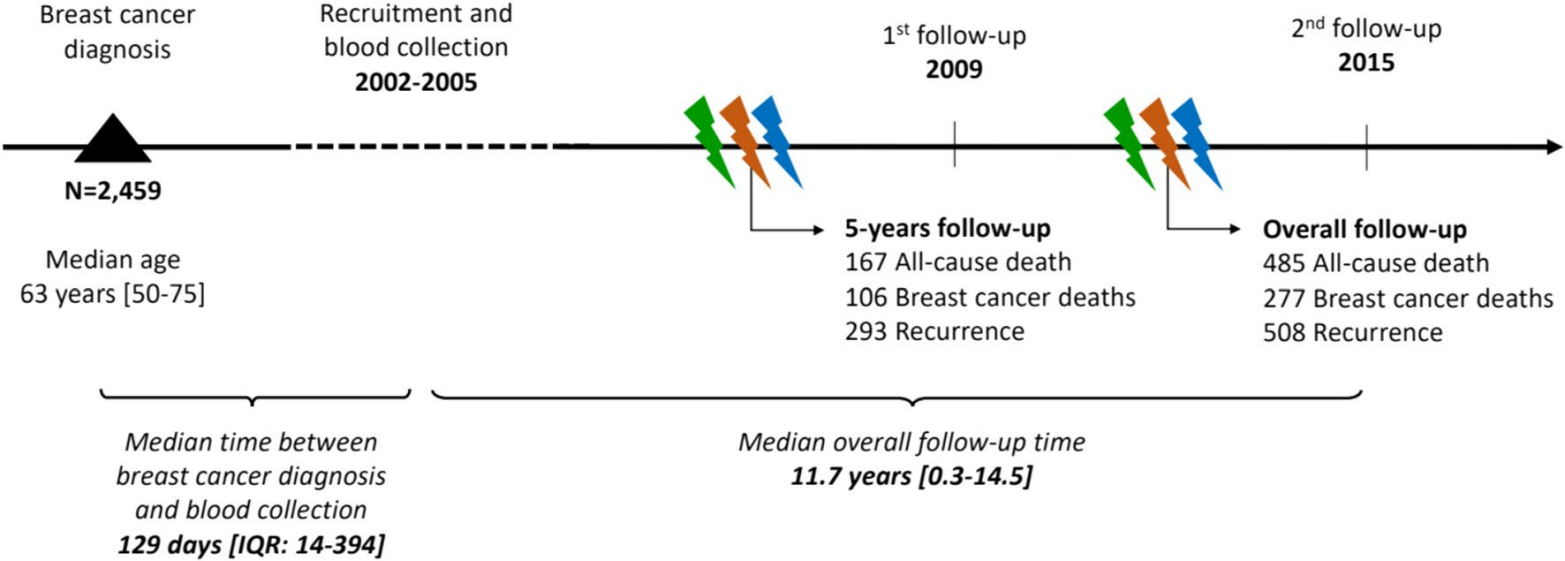
Study timeline from inclusion to the last follow-up

Assays were conducted on blood samples collected from participants at recruitment (median 129 days [interquartile range: 14–394] after breast cancer diagnosis) (Fig. 2). A subsample of 28 women had OPG and TRAIL measured additionally on a subsequent blood sample collected in 2009; these samples were used to assess within-person stability. Serum concentrations of OPG and TRAIL were analyzed at the Laboratory of the Division of Cancer Epidemiology at the German Cancer Research Center using electrochemiluminescence assays and the MESO Quickplex SQ 120 system from Meso Scale Discoveries (Gaithersburg, MD). Stock kits were purchased for the measurement both of human TRAIL (pg/ml) and OPG (pg/ml), and the samples were measured according to the manufacturer’s recommendations. All samples were measured in a blinded fashion with quality control samples measured in duplicate across all batches. The coefficient of variation (CV) within each batch was 8.1% for OPG and 4.9% for TRAIL. Between batches, the CVs for OPG and TRAIL were 15.2% and 15.1%, respectively.

### Covariate assessment

Self-reported information about lifestyle, demographic and socioeconomic characteristics, and comorbidities and treatment modalities was collected from the baseline interview, as previously described [14]. Clinical and pathology data were obtained from hospital and pathology records.

### Outcome assessment

The vital status of participants was retrieved through central population registry databases from the study regions up to the end of June 2015, and all deaths were verified by death certificates from local health offices. Causes of death were coded according to the 10th revision of the International Classification of Diseases (ICD-10). Self-reported recurrences of the primary breast cancer, second cancers, and metastatic disease were provided at two follow-ups (2009, 2015) were verified by clinical records or with treating physicians. For patients who died, data were collected from medical records or treating physicians. Primary outcomes were all-cause mortality (57% breast cancer, 18% other cancers, 13% cardiovascular disease, and 12% other causes), breast cancer-specific mortality, and recurrence (defined as: ipsilateral/local/regional invasive recurrence, distant recurrence and metastases occurring after primary diagnosis, or any death) [15]. Evaluations of recurrence were restricted to participants diagnosed with breast cancer stage 0-IIIa, having blood sample collected before any type of recurrence, and recurrence status known (Fig. 1).

### Statistical analysis

OPG and TRAIL were log2-transformed for analyses on the continuous scale and categorized into quartiles based on the distribution in the full study sample. Linear regression models adjusted for age and study center were used to assess cross-sectional associations between OPG and TRAIL concentrations and clinical and lifestyle characteristics. Pearson partial correlations adjusted for age were calculated for the subsample of 28 women with OPG and TRAIL measured at two time points (median time between blood collections: 5.2 years (range: 3.6–5.9)) to estimate within-person stability of analyte levels.

Delayed-entry Cox proportional hazards regression was used to estimate hazard ratios (HRs) and corresponding 95% confidence intervals (CIs) for associations between OPG and TRAIL with outcomes overall and by tumor hormone receptor subtype. Analyses by hormone receptor subtype are restricted to invasive disease. Time-to-event started from the date of diagnosis, and time-at-risk started from the date of blood collection. Years from diagnosis was used as the timescale. Women who did not experience one of the defined endpoints (recurrence or death) after diagnosis were censored at the time of the last follow-up. Associations were investigated for the entire duration of follow-up (median = 11.7 (range = 0.30–14.5) years calculated using reverse Kaplan–Meier method [16]), as well as considering 5-year survival as a secondary analysis. Deviations from linearity were tested by comparing the model with the linear term only to the model with the linear and the cubic spline terms [17].

Heterogeneity in associations between OPG and TRAIL and outcomes by tumor hormone receptor subtype at diagnosis (ER + PR + , discordant (ER + /PR- or ER-/PR +), ER-/PR-) were assessed by evaluating the Wald p-value associated with an interaction term in the model. Heterogeneity by HER2 status, and restriction to triple-negative tumors, was also investigated. Additionally, we assessed heterogeneity by BMI (< 25, ≥ 25 kg/m2) and conducted a sensitivity analysis restricted to women reporting natural menopause (91%).

All models were adjusted for the following prognostic factors: tumor size, nodal status, tumor grade, and age, and stratified by study center and hormone receptor status. The proportional hazards assumption was evaluated using a weighted least‐squares line fitted to the plots of scaled Schoenfeld residuals [18]; no violations were observed. Potential further covariates including time between operation and blood collection, time between chemotherapy and blood collection, mode of tumor detection, use of chemotherapy, radiotherapy, SERM or AI, type of surgery, HER2 status, menopausal hormone use at diagnosis, body mass index (BMI), physical activity, education, alcohol consumption, smoking, comorbidities (cardiovascular disease, diabetes), and laboratory batch were evaluated for statistical adjustment. The inclusion of additional adjustment factors did not change the HRs by more than 10%, and thus these variables were not included in the final models. The proportion of missing values was less than 5% for all covariates, and complete-case analysis was performed.

In two sensitivity analyses, we excluded women who used neoadjuvant chemotherapy, were diagnosed with tumors stage 0 (in situ*) or* IIIb or higher, and who had blood drawn (1) within 7 days or (2) within 3 months after surgery. The cutoff points for the timing of blood collection were selected based on data showing OPG concentrations tended to be lower up to 3 months after surgery (Additional file 1: Table S1, figure S1) as compared to concentrations in samples collected more distant from surgery. Potential reverse causation was investigated by excluding women with recurrence less than 2 years after blood was drawn (n = 156).

All statistical tests were two-sided, and the significance level was set to 0.05. Analyses were conducted using SAS statistical software package (version 9.4).

## Results

A total of 2,459 total breast cancer patients (93.7% invasive disease) were included in this study (Fig. 1), with a median age at breast cancer diagnosis of 62.9 years [range: 49.6–75.0]. The median follow-up time of study participants was 11.7 years. During follow-up, a total of 485 women (19.7%) died from any cause, 277 (11.3%) died from breast cancer, and 508 (25%) women developed a recurrence (among women with tumor stage below IIIb at diagnosis). At 5 years after diagnosis, 167 (7%) women died, 106 (4%) died from breast cancer, and 293 (12%) women developed a recurrence (Fig. 2).

In the cross-sectional analysis, older age was associated with higher circulating OPG and TRAIL concentrations (Table 1), and higher concentrations of OPG were observed among women with higher BMI, those reporting no alcohol consumption, and those reporting diabetes or history of cardiovascular disease (Additional file 1: Table S1). TRAIL was further inversely associated with tumor stage, with higher concentrations in women diagnosed with stage 0 breast cancer or carcinoma in situ, and HER2 + breast cancer molecular subtype. Higher concentrations of TRAIL were also observed in women who did not report chemotherapy, and who were not current smokers (never and former). No other associations were observed between OPG or TRAIL and further clinical or treatment characteristics or lifestyle factors reported (e.g., radiation therapy, menopausal status).

**Table 1:** Geometric means for OPG and TRAIL (in pg/ml) adjusted for age and center by clinical characteristics

	n = 2,456 n (%)	OPG Geometric mean (95%CI)	*p*^a^	n = 2,457 n (%)	TRAIL Geometric mean (95%CI)	*p*^a^
*Age at diagnosis (years)*^*b*^						
50–54 55–59 60–64 65–69 70–75	374 (15.2) 499 (20.3) 720 (29.3) 612 (24.9) 251 (10.2)	175.1 (169.1–181.4) 192.6 (186.9–198.6) 204.6 (199.5–209.8) 215.4 (209.6–221.4) 239.0 (229.0–249.5)	< 0.0001	375 (15.3) 501 (20.4) 719 (29.3) 611 (24.9) 251 (10.2)	18.2 (17.3–19.1) 19.8 (19.0–20.7) 20.4 (19.6–21.1) 20.5 (19.7–21.3) 20.1 (18.9–21.4)	0.003
*Stage*^*c*^						
In situ/stage 0	154 (6.5)	203.0 (192.3–214.3)	0.90	154 (6.5)	22.2 (20.5–24.1)	0.01
0-IIIa	2104 (89.0)	203.2 (200.2–206.2)		2106 (89.0)	19.8 (19.4–20.2)	
≥ IIIb	107 (4.5)	206.4 (193.4–220.3)		106 (4.5)	20.9 (19.0–23.0)	
*Tumor size (cm)*^*c,d*^						
≤ 2	1303 (58.9)	201.9 (198.1–205.7)	0.52	1303 (58.9)	19.8 (19.3–20.3)	0.90
> 2–5	787 (35.6)	206.0 (201.1–211.0)		788 (35.6)	20.0 (19.3–20.7)	
> 5	77 (3.5)	209.3 (193.9–226.1)		77 (3.5)	19.3 (17.2–21.6)	
Growth into chest wall	44 (2.0)	200.6 (181.2–222.0)		44 (2.0)	20.4 (17.6–23.7)	
*Nodal status*^*c,d*^						
0	1529 (69.2)	202.5 (199.1–206.1)	0.10	1529 (69.1)	20.0 (19.5–20.5)	0.64
1–3	486 (22.0)	202.6 (196.5–208.9)		487 (22.0)	19.6 (18.8–20.5)	
≥ 4	196 (8.9)	214.1 (204.0–224.7)		196 (8.9)	19.4 (18.1–20.8)	
*Tumor grade*^*c,d*^						
Low/Moderate	1637 (74.0)	202.6 (199.3–206.0)	0.30	1638 (74.1)	20.0 (19.5–20.5)	0.26
High	574 (26.0)	206.2 (200.4–212.1)		574 (26.0)	19.5 (18.7–20.3)	
*Hormone receptor status*^*c,d*^						
ER + PR +	1535 (69.4)	201.9 (198.5–205.5)	0.09	1536 (69.4)	19.8 (19.3–20.3)	0.49
ER + /PR-/ER-PR + /hormone therapy	367 (16.6)	203.5 (196.5–210.8)		367 (16.6)	19.6 (18.6–20.6)	
ER-PR-	309 (14.0)	211.7 (203.7–219.9)		309 (14.0)	20.4 (19.3–21.6)	
*HER2 status*^*c,d*^						
HER2 +	440 (21.0)	205.2 (198.7–211.8)	0.70	440 (21.0)	21.2 (20.2–22.2)	0.003
HER2-	1656 (79.0)	203.7 (200.4–207.1)		1656 (79.0)	19.6 (19.1–20.1)	

*OPG* osteoprotegerin; *ER* estrogen receptor; *n* number of observations; *p* p-value/probability; *PR* progesterone receptor; *TRAIL* TNF-related apoptosis-inducing ligand

^a^Cross-sectional associations between clinical characteristics and OPG and TRAIL were evaluated in a linear regression model adjusted for age and center

^b^Age at diagnosis is adjusted for center only

^c^Additionally 91 women had neoadjuvant chemotherapy and no pre-treatment data available

^d^Additionally 154 women were diagnosed tumor in situ or stage 0

Missing: HER2 status n = 115 (OPG) n = 116 (TRAIL)

Concentrations were lower in women with blood collection within less than 3 months of breast cancer surgery for OPG, and within 7 days of surgery for TRAIL, compared to blood collected later (Additional file 1: Table S1, figure S1). The Pearson age-adjusted partial correlation between log2-transformed concentrations of OPG and TRAIL at baseline was 0.17, and the correlation of concentrations in blood samples taken at baseline and after 5 years of follow-up was 0.28 for OPG and 0.53 for TRAIL.

Higher OPG was associated with a higher risk of all-cause mortality (HR, 1 unit increase in log2-transformed OPG (HR_log2_): 1.24 (1.03–1.49)) for all cases (Table 2). While there was no statistically significant heterogeneity by hormone receptor status (p for heterogeneity by disease subtype (p_het_) = 0.09), associations were only observed for ER-PR- and ERPR-discordant disease (ER-PR-, 1.93 (1.20–3.10); ERPR-discordant, 1.70 (1.03–2.81), ER + PR + , 1.06 (0.83–1.35)). Higher circulating OPG was not significantly associated with a higher risk of breast cancer-specific death but was associated with a higher risk of recurrence among cases diagnosed with ER-PR- disease (HR_log2_: 2.18 (95% CI: 1.39–3.40); p_het_ = 0.01). No significant associations for recurrence were observed for ERPR-discordant or ER + PR + cases, though the HR for the ERPR-discordant group (HR_log2_: 1.50 (0.92–2.45)) was intermediate to the ER-PR- (HR_log2_: 2.18 (1.39–3.40)) and ER + PR + groups (HR_log2_: 0.97 (0.77–1.21)). Associations in triple-negative cases were similar to those for ER-PR- cases (HR_log2_: 1.97 (1.16–3.34); data not tabled). We observed no heterogeneity in associations by HER2 status. Associations for 5-year survival followed a similar pattern to those observed for the full follow-up period, though HRs were somewhat attenuated for some associations (e.g., ER-PR-, HR_log2_ full follow-up: 1.93 (1.20–3.10); 5-year follow-up: 1.55 (0.79–3.06) (Additional file 1: Table S2).

**Table 2:** Hazard ratios and corresponding 95% confidence intervals for the association between circulating OPG concentration at blood collection and survival outcomes, stratified by ERPR status

		OPG, all cases					ER-PR-					ER+ PR− or ER-PR +					ER + PR +				
		n/events	HR^a^	(95% CI)	*p*		n/events	HR^b^	(95% CI)	*p*		n/events	HR^b^	(95% CI)	*p*		n/events	HR^b^	(95% CI)	*p*	Phet^c^
*All-cause mortality*																					
Quartiles	1	613/108		﻿ref.			68/14		﻿ref.			88/15		﻿ref.			396/68		﻿ref.		
	2	614/102	0.85	(0.65–1.12)			79/16	0.94	(0.45–1.99)			103/20	1.08	(0.54–2.13)			370/56	0.84	(0.59–1.20)		0.44
	3	615/122	0.97	(0.75–1.27)			69/22	1.40	(0.70–2.82)			88/16	0.89	(0.43–1.85)			392/72	0.96	(0.68–1.35)		
	4	614/152	1.21	(0.93–1.56)			93/34	1.75	(0.90–3.40)			88/23	1.58	(0.81–3.06)			377/85	1.09	(0.78–1.52)		
Continuous^d^		2456/484	1.24	(1.03–1.49)	0.03		309/86	1.93	(1.20–3.10)	0.01		367/74	1.70	(1.03–2.81)	0.04		1535/281	1.06	(0.83–1.35)	0.64	0.09
*Breast cancer-specific mortality*																					
Quartiles	1	613/72		﻿ref.			68/10		﻿ref.			88/10		﻿ref.			396/44		﻿ref.		
	2	614/55	0.71	(0.50–1.02)			79/12	1.11	(0.46–2.69)			103/14	1.20	(0.52–2.76)			370/24	0.56	(0.34–0.92)		0.48
	3	615/71	0.86	(0.62–1.21)			69/15	1.40	(0.60–3.27)			88/11	1.04	(0.43–2.53)			392/35	0.75	(0.48–1.18)		
	4	614/78	0.97	(0.70–1.36)			93/18	1.33	(0.58–3.09)			88/15	1.66	(0.73–3.77)			377/40	0.80	(0.51–1.26)		
Continuous^d^		2456/276	1.08	(0.84–1.39)	0.53		309/55	1.37	(0.75–2.48)	0.30		367/50	1.78	(0.96–3.30)	0.07		1535/143	0.92	(0.66–1.29)	0.63	0.09
*Recurrence-free survival*^*e*^																					
Quartiles	1	558/117		﻿ref.			62/13		﻿ref.			83/18		﻿ref.			376/83		﻿ref.		
	2	566/116	0.92	(0.71–1.20)			75/20	1.32	(0.64–2.73)			99/20	0.86	(0.45–1.65)			353/71	0.86	(0.63–1.19)		0.05
	3	555/129	1.09	(0.84–1.40)			63/22	1.93	(0.94–3.97)			81/17	0.89	(0.45–1.76)			371/86	0.99	(0.73–1.34)		
	4	560/145	1.13	(0.88–1.46)			88/37	2.35	(1.20–4.58)			84/23	1.31	(0.70–2.45)			352/83	0.93	(0.67–1.27)		
Continuous^d^		2239/507	1.16	(0.97–1.39)	0.11		288/92	2.18	(1.39–3.40)	< 0.001		347/78	1.50	(0.92–2.45)	0.11		1452/323	0.97	(0.77–1.21)	0.77	0.01

ERPR status was not available for 91 women who had neoadjuvant chemotherapy and 154 who were diagnosed tumor in situ or stage 0. ERPR-discordant includes ER+PR-, ER-PR+, ER+ or PR+ and data on other receptor unknown, or women treated with tamoxifen or aromatase inhibitor

^a^Adjusted for age, nodal status, tumor size, and grade/ strata for center, ERPR status

^b^Adjusted for age, nodal status, tumor size, and grade/ strata for center

^c^Heterogeneity of estimates across ERPR status

^d^Log2 transformed

^e^Patients without information on recurrence or with a tumor stages 3b, and 3c were excluded

There was no evidence of an association between TRAIL and any outcome in both the full follow-up (Table 3) and at 5 years (Additional file 1: Table S3). Overall, there was no association between TRAIL and breast cancer mortality and recurrence-free survival.

**Table 3:** Hazard ratios and corresponding 95% confidence intervals for the association between circulating TRAIL concentration at blood collection and survival outcomes, stratified by ERPR status

		TRAIL, all cases				ER-PR-				ER+ PR− or ER-PR +				ER + PR +				
		n/events	HR^a^	(95% CI)	*p*	n/events	HR^b^	(95% CI)	*p*	n/events	HR^b^	(95% CI)	*p*	n/events	HR^b^	(95% CI)	*p*	Phet^c^
*All-cause mortality*																		
Quartiles	1	614/132		ref.		66/21		﻿ref.		99/19		﻿ref.		387/77		﻿ref.		
	2	614/111	0.80	(0.62–1.03)		78/22	0.79	(0.43–1.46)		93/18	0.94	(0.49–1.80)		385/61	0.79	(0.57–1.11)		0.71
	3	615/124	0.93	(0.73–1.19)		81/24	0.94	(0.51–1.73)		83/20	1.29	(0.68–2.45)		393/74	0.91	(0.66–1.26)		
	4	614/118	0.86	(0.67–1.11)		84/20	0.71	(0.38–1.32)		92/17	1.00	(0.51–1.96)		371/69	0.89	(0.64–1.23)		
Continuous^d^		2457/485	0.98	(0.87–1.11)	0.80	309/87	0.98	(0.73–1.31)	0.88	367/74	1.09	(0.81–1.46)	0.74	1536/281	0.96	(0.81–1.13)	0.62	0.67
*Breast cancer-specific mortality*																		
Quartiles	1	614/66		﻿ref.		66/11		﻿ref.		99/14		﻿ref.		387/34		﻿ref.		
	2	614/60	0.90	(0.64–1.29)		78/16	1.11	(0.51–2.42)		93/12	0.78	(0.36–1.72)		385/26	0.86	(0.51–1.45)		0.48
	3	615/83	1.35	(0.97–1.87)		81/18	1.47	(0.67–3.24)		83/14	1.15	(0.54–2.44)		393/46	1.41	(0.90–2.20)		
	4	614/68	1.07	(0.76–1.50)		84/11	0.80	(0.34–1.87)		92/10	0.84	(0.36–1.95)		371/37	1.16	(0.72–1.86)		
Continuous^d^		2457/277	1.10	(0.93–1.30)	0.26	309/56	1.06	(0.73–1.54)	0.76	367/50	0.98	(0.68–1.43)	0.96	1536/143	1.09	(0.86–1.38)	0.49	0.81
*Recurrence-free survival*^*e*^																		
Quartiles	1	546/133		﻿ref.		61/21		﻿ref.﻿		92/22		﻿ref.		365/85		﻿ref.		
	2	566/118	0.81	(0.63–1.04)		71/23	0.94	(0.52–1.72)		92/20	0.78	(0.42–1.46)		369/70	0.79	(0.57–1.08)		0.80
	3	570/136	0.98	(0.77–1.24)		77/25	0.97	(0.54–1.76)		80/21	1.04	(0.56–1.90)		371/89	1.04	(0.77–1.41)		
	4	559/121	0.89	(0.69–1.14)		79/24	0.97	(0.54–1.76)		83/15	0.79	(0.40–1.53)		349/79	0.93	(0.68–1.27)		
Continuous^d^		2241/508	1.00	(0.88–1.13)	0.96	288/93	1.09	(0.82–1.46)	0.56	347/78	0.96	(0.72–1.28)	0.71	1454/323	1.01	(0.87–1.18)	0.88	0.88

ERPR status was not available for 91 women who had neoadjuvant chemotherapy and 154 who were diagnosed tumor in situ or stage 0. ERPR-discordant includes ER+PR-, ER-PR+, ER+ or PR+ and data on other receptor unknown, or women treated with tamoxifen or aromatase inhibitor

^a^Adjusted for age, nodal status, tumor size, and grade/strata for center, ERPR status

^b^Adjusted for age, nodal status, tumor size, and grade/strata for center

^c^Heterogeneity of estimates across ERPR status

^d^Log-2-transformed

^e^Patients without information on recurrence or with a tumor stages 3b, and 3c were excluded

In analyses stratified by BMI, a positive association was observed between OPG and all-cause mortality in women with BMI ≥ 25 kg/m^2^ (HR_log2:_ 1.48 (1.17–1.88)) but not in women with BMI < 25 kg/m^2^ (HR_log2:_ 0.87 (0.64–1.18); p_het_ = 0.02). When restricting the analysis to women reporting natural menopause, associations were similar to those observed in the full study sample. No differences by BMI or menopausal status were observed for associations between TRAIL with mortality and recurrence.

Finally, we conducted sensitivity analyses excluding women based on the stage at diagnosis, use of neoadjuvant chemotherapy, and the time between surgery and blood collection. The pattern of associations for OPG was similar to that observed in our primary analyses (Additional file 1: Table S4; Table S5). No associations were observed between TRAIL and any outcome over the full follow-up period, though select associations were observed at 5 years of follow-up (e.g., excluding women with blood drawn within 3 months after breast surgery (breast cancer-specific mortality (HRlog2: 1.93 (1.04–3.56); recurrence-free survival (HRlog2: 1.39 (1.00–1.93), Additional file 1: Table S4). Findings were similar in the analyses evaluating reverse causation and excluding outcomes in the first 2 years of follow-up.

## Discussion

In this large, well-characterized patient cohort, we observed associations between circulating OPG and survival following a breast cancer diagnosis, with associations predominantly observed among women with hormone receptor-negative tumors, or those with tumors with discordant hormone receptor status and presumably not fully hormone responsive. Circulating TRAIL concentrations were not associated with any of the evaluated outcomes, except in select subgroup analyses. These data add to the limited existing data on these members of the broader RANK signaling pathway and breast cancer survival.

OPG has an established role as a decoy receptor for TRAIL and RANKL, inhibiting receptor binding and consequent activation of downstream signaling pathways. The canonical actions of TRAIL are mediated via cell surface receptors TRAIL-R1 and -R2 (also known as death receptors (DR)4 and DR5) with intracellular death domains and activation of an apoptotic program. Further non-apoptosis-inducing TRAIL receptors include TRAIL-R3 and -R4, in addition to OPG [19–21]. OPG has been demonstrated to have tumor-promoting effects in breast cancer, hypothesized to be mediated in part through its role as a decoy receptor for TRAIL [22]. RANKL-RANK signaling plays a key role in bone homeostasis, including in the regulation of bone metabolism in metastases, and RANKL-RANK binding promotes mammary cell proliferation and survival [22]. OPG as a decoy receptor for RANKL would be hypothesized to have an inhibitory effect on mammary cancer progression, as opposed to OPG as a potential cancer promoter given its interactions with TRAIL.

This study is the first to evaluate circulating OPG and TRAIL and outcomes following a breast cancer diagnosis in a large cohort of breast cancer patients with postdiagnosis blood samples (n = 2,456; largest prior study in breast cancer patients, n = 504). We observed a higher risk of overall death, but not breast cancer-specific death, with higher OPG in the current study. To our knowledge, only two studies have evaluated circulating OPG and breast cancer survival, with one study measuring circulating postdiagnosis OPG in breast cancer patients (median postdiagnosis follow-up, 8.5 years) [8], as in the current study, and one, in the EPIC cohort, evaluating pre-diagnosis circulating OPG in women who were subsequently diagnosed with breast cancer (median time between blood collection and later breast cancer diagnosis, 4.7 years, median postdiagnosis follow-up, 10.9 years) [9]. The most recent and comparable study, among 504 breast cancer patients, observed significantly worse breast cancer-specific survival among women with relatively high serum OPG concentrations after diagnosis (above vs. below median, RR = 1.70 (1.04–2.80)) [8]. In the EPIC cohort, higher pre-diagnosis OPG concentrations among 2006 cases were associated with a higher risk of death following a breast cancer diagnosis [9]. One further study in a general population-based cohort evaluated pre-diagnosis circulating OPG and overall cancer-related mortality (i.e., death from any cancer) [23] and showed that higher OPG concentrations were associated with a higher risk of cancer-related mortality (per 1-standard deviation increase in OPG, HR = 1.25 (1.11–1.39)). Breast cancer mortality was not evaluated as a separate outcome in this prior study. These findings are generally in line with the current results in women with a breast cancer diagnosis.

Associations between OPG and mortality following a breast cancer diagnosis were only observed among participants with ER-PR- and ERPR-discordant tumors in the current study. While no significant associations were observed for breast cancer-specific mortality, higher OPG was associated with a higher risk of recurrence among women with ER-PR- tumors. Prior studies on circulating OPG and survival following a breast cancer diagnosis have largely investigated associations in hormone receptor-positive disease [8, 9], given that this is the predominant subtype. In the EPIC cohort study on pre-diagnosis concentrations and breast cancer survival, no heterogeneity by ER status was observed, and while associations were only observed in ER + cases (n = 1620), the number of ER- cases was limited (n = 386) [9]. Intriguingly, OPG was associated with a higher risk of ER- disease in that cohort [5]. Prior studies on tumor OPG and survival following a breast cancer diagnosis have evaluated protein and mRNA expression in the tumor tissue, though findings are equivocal and with no clear patterns by tumor hormone receptor status (reviewed in [7]).

The associations observed in women with hormone receptor-negative disease in the current study were in line with our a priori hypothesis for OPG, given data suggesting that TRAIL-induced apoptosis is particularly relevant in hormone receptor-negative breast cancer cell lines [10–12] and that OPG decreases TRAIL-induced apoptosis in these cell lines [12, 13]. However, contrary to our initial hypotheses, TRAIL was not associated with any of the evaluated outcomes in this study. Potential confounding and/or treatment-related factors such as chemotherapy or smoking status were associated with circulating TRAIL concentrations in this study, but adjustment for these factors did not change the magnitude of the associations between TRAIL and recurrence or survival. We measured TRAIL in circulation, and it is plausible that concentrations in circulation are not reflective of concentrations in the relevant local tissue(s) implicated in breast cancer progression. Additionally, given the opposing action of cell surface receptors subtypes of TRAIL (R1, R2 apoptotic and R3, R4 anti-apoptotic), future research is warranted.

The lack of data on OPG and TRAIL, and TRAIL receptors, at the tumor tissue level is a limitation of this study. Further studies characterizing circulating and tumor tissue levels are needed to inform the interpretation of the associations observed here and previously. Further limitations include the single measurement of OPG and TRAIL, which may not reflect longer-term concentrations. Based on the data available to date, the relevant window of exposure for prognosis is not established (e.g., proximate or distant to diagnosis). The current study included samples collected at median of 129 days after breast cancer diagnosis and during time of active treatment, and while treatment data were collected and had minimal impact on concentrations, concentrations collected more proximate to diagnosis and before active treatment may be of relevance.

We observed that higher circulating OPG may be a biomarker of a higher risk of poor outcome among women diagnosed with ER-PR- or ER/PR-discordant breast cancer. While further mechanistic studies are needed, OPG may be a marker of prognosis in breast cancer patients.

## Supplementary Information

**Additional file1**. Postdiagnosis circulating osteoprotegerin and TRAIL concentrations and survival and recurrence after a breast cancer diagnosis: Results from the MARIE patient cohort: Tables S1- S5 and Figure S1. **Table S1**: Geometric means for OPG and TRAIL (in pg/mL) adjusted for age and center by clinical characteristics and lifestyle factors. **Table S2**: Hazard ratios and corresponding 95% confidence intervals for the association between circulating OPG concentration at blood collection and 5-years survival, stratified by ERPR status. **Table S3**: Hazard ratios and corresponding 95% confidence intervals for the association between circulating TRAIL concentration at blood collection and 5-years survival, stratified by ERPR status. **Table S4**: Sensitivity analysis for the association between circulating OPG and TRAIL concentration at blood collection and overall survival. **Table S5**: Sensitivity analysis for the association between circulating OPG and TRAIL concentration at blood collection and 5-years survival. Figure S1: OPG and TRAIL concentrations by time between breast cancer operation and blood collection.

## Data Availability

The data can be made available upon reasonable request to the principal investigator(s) (JCC and HB).

## References

[1] Gonzalez-Suarez E, Jacob AP, Jones J, Miller R, Roudier-Meyer MP, Erwert R, Pinkas J, Branstetter D, Dougall WC (2010). RANK ligand mediates progestin-induced mammary epithelial proliferation and carcinogenesis. Nature.

[2] Schramek D, Leibbrandt A, Sigl V, Kenner L, Pospisilik JA, Lee HJ, Hanada R, Joshi PA, Aliprantis A, Glimcher L (2010). Osteoclast differentiation factor RANKL controls development of progestin-driven mammary cancer. Nature.

[3] Joshi PA, Jackson HW, Beristain AG, Di Grappa MA, Mote PA, Clarke CL, Stingl J, Waterhouse PD, Khokha R (2010). Progesterone induces adult mammary stem cell expansion. Nature.

[4] Asselin-Labat ML, Vaillant F, Sheridan JM, Pal B, Wu D, Simpson ER, Yasuda H, Smyth GK, Martin TJ, Lindeman GJ (2010). Control of mammary stem cell function by steroid hormone signalling. Nature.

[5] Fortner RT, Sarink D, Schock H, Johnson T, Tjonneland A, Olsen A, Overvad K, Affret A, His M, Boutron-Ruault MC (2017). Osteoprotegerin and breast cancer risk by hormone receptor subtype: a nested case-control study in the EPIC cohort. BMC Med.

[6] Kotsopoulos J, McGee EE, Lozano-Esparza S, Garber JE, Ligibel J, Collins LC, Polyak K, Brown M, Narod S, Tamimi RM (2020). Premenopausal plasma osteoprotegerin and breast cancer risk: a case-control analysis nested within the nurses' health study II. Cancer Epidemiol Biomark Prev.

[7] Geerts D, Chopra C, Connelly L (2020). Osteoprotegerin: relationship to breast cancer risk and prognosis. Front Oncol.

[8] Rachner TD, Kasimir-Bauer S, Gobel A, Erdmann K, Hoffmann O, Browne A, Wimberger P, Rauner M, Hofbauer LC, Kimmig R (2019). Prognostic value of RANKL/OPG serum levels and disseminated tumor cells in nonmetastatic breast cancer. Clin Cancer Res.

[9] Sarink D, Schock H, Johnson T, Chang-Claude J, Overvad K, Olsen A, Tjonneland A, Arveux P, Fournier A, Kvaskoff M (2018). Receptor activator of nuclear factor kB ligand, osteoprotegerin, and risk of death following a breast cancer diagnosis: results from the EPIC cohort. BMC Cancer.

[10] Rahman M, Pumphrey JG, Lipkowitz S (2009). The TRAIL to targeted therapy of breast cancer. Adv Cancer Res.

[11] Zinonos I, Labrinidis A, Lee M, Liapis V, Hay S, Ponomarev V, Diamond P, Findlay DM, Zannettino AC, Evdokiou A (2011). Anticancer efficacy of Apo2L/TRAIL is retained in the presence of high and biologically active concentrations of osteoprotegerin in vivo. J Bone Miner Res.

[12] Neville-Webbe HL, Cross NA, Eaton CL, Nyambo R, Evans CA, Coleman RE, Holen I (2004). Osteoprotegerin (OPG) produced by bone marrow stromal cells protects breast cancer cells from TRAIL-induced apoptosis. Breast Cancer Res Treat.

[13] Holen I, Cross SS, Neville-Webbe HL, Cross NA, Balasubramanian SP, Croucher PI, Evans CA, Lippitt JM, Coleman RE, Eaton CL (2005). Osteoprotegerin (OPG) expression by breast cancer cells in vitro and breast tumours in vivo—a role in tumour cell survival?. Breast Cancer Res Treat.

[14] Flesch-Janys D, Slanger T, Mutschelknauss E, Kropp S, Obi N, Vettorazzi E, Braendle W, Bastert G, Hentschel S, Berger J (2008). Risk of different histological types of postmenopausal breast cancer by type and regimen of menopausal hormone therapy. Int J Cancer.

[15] Hudis CA, Barlow WE, Costantino JP, Gray RJ, Pritchard KI, Chapman JA, Sparano JA, Hunsberger S, Enos RA, Gelber RD (2007). Proposal for standardized definitions for efficacy end points in adjuvant breast cancer trials: the STEEP system. J Clin Oncol.

[16] Schemper M, Smith TL (1996). A note on quantifying follow-up in studies of failure time. Control Clin Trials.

[17] Durrleman S, Simon R (1989). Flexible regression models with cubic splines. Stat Med.

[18] Grambsch PM, Therneau TM (1994). Proportional hazards tests and diagnostics based on weighted residuals. Biometrika.

[19] Bertsch U, Roder C, Kalthoff H, Trauzold A (2014). Compartmentalization of TNF-related apoptosis-inducing ligand (TRAIL) death receptor functions: emerging role of nuclear TRAIL-R2. Cell Death Dis.

[20] Emery JG, McDonnell P, Burke MB, Deen KC, Lyn S, Silverman C, Dul E, Appelbaum ER, Eichman C, DiPrinzio R (1998). Osteoprotegerin is a receptor for the cytotoxic ligand TRAIL. J Biol Chem.

[21] Kimberley FC, Screaton GR (2004). Following a TRAIL: update on a ligand and its five receptors. Cell Res.

[22] Infante M, Fabi A, Cognetti F, Gorini S, Caprio M, Fabbri A (2019). RANKL/RANK/OPG system beyond bone remodeling: involvement in breast cancer and clinical perspectives. J Exp Clin Cancer Res.

[23] Vik A, Brodin EE, Mathiesen EB, Brox J, Jorgensen L, Njolstad I, Braekkan SK, Hansen JB (2015). Serum osteoprotegerin and future risk of cancer and cancer-related mortality in the general population: the Tromso study. Eur J Epidemiol.

