# Supplementary material for: Postdiagnosis circulating osteoprotegerin and TRAIL concentrations and survival and recurrence after a breast cancer diagnosis: results from the MARIE patient cohort
Source: Breast Cancer Res. 2023 Apr 17;25:42. doi: 10.1186/s13058-023-01625-4 (PMC10108482; doi:10.1186/s13058-023-01625-4)
Supplement: Supplementary file 1 — Additional file1. Postdiagnosis circulating osteoprotegerin and TRAIL concentrations and survival and recurrence after a breast cancer diagnosis: Results from the MARIE patient cohort: Tables S1- S5 and Figure S1. Table S1: Geometric means for OPG and TRAIL (in pg/mL) adjusted for age and center by clinical characteristics and lifestyle factors. Table S2: Hazard ratios and corresponding 95% confidence intervals for the association between circulating OPG concentration at blood collection and 5-years survival, stratified by ERPR status. Table S3: Hazard ratios and corresponding 95% confidence intervals for the association between circulating TRAIL concentration at blood collection and 5-years survival, stratified by ERPR status. Table S4: Sensitivity analysis for the association between circulating OPG and TRAIL concentration at blood collection and overall survival. Table S5: Sensitivity analysis for the association between circulating OPG and TRAIL concentration at blood collection and 5-years survival. Figure S1: OPG and TRAIL concentrations by time between breast cancer operation and blood collection. [file 13058_2023_1625_MOESM1_ESM.docx]

**Additional file 1: Postdiagnosis circulating osteoprotegerin and TRAIL concentrations and survival and recurrence after a breast cancer diagnosis: Results from the MARIE patient cohort: Supplementary Tables S1- S5 and Figure S1.**

**Table S1**. Geometric means for OPG and TRAIL (in pg/mL) adjusted for age and center by clinical characteristics and lifestyle factors.

|  | **n = 2,456** | **OPG** | **p**^a^ | **n = 2,457** | **TRAIL** | **p**^a^ |
| --- | --- | --- | --- | --- | --- | --- |
|  | **n (%)** | **Geometric mean (95%CI)** |  | **n (%)** | **Geometric mean (95%CI)** |  |
| **Mode of tumor detection^b^** |  |  |  |  |  |  |
| Self-detected | 1292 (52.8) | 204.8 (200.9-208.7) | 0.22 | 1292 (52.8) | 19.9 (19.3-20.4) | 0.72 |
| Routine examination | 1156 (47.2) | 201.3 (197.3-205.3) |  | 1157 (47.2) | 20.0 (19.4-20.6) |  |
| **Chemotherapy** |  |  |  |  |  |  |
| Yes | 1154 (47.5) | 203.1 (199.1-207.3) | 0.94 | 1154 (47.5) | 19.5 (19.0-20.1) | 0.04 |
| No | 1276 (52.5) | 203.3 (199.5-207.3) |  | 1277 (52.5) | 20.4 (19.8-21.0) |  |
| **Radiation therapy** |  |  |  |  |  |  |
| Yes | 1910 (78.5) | 204.5 (201.4-207.7) | 0.08 | 1909 (78.4) | 20.1 (19.6-20.6) | 0.23 |
| No | 523 (21.5) | 198.5 (192.8-204.5) |  | 525 (21.6) | 19.5 (18.7-20.4) |  |
| **Tamoxifen / aromatase inhibitor use** |  |  |  |  |  |  |
| Yes | 1916 (81.3) | 201.9 (198.8-205.0) | 0.20 | 1916 (81.3) | 19.8 (19.4-20.3) | 0.11 |
| No | 441 (18.7) | 206.7 (200.2-213.4) |  | 442 (18.7) | 20.7 (19.7-21.7) |  |
| **Bisphosphonate therapy** |  |  |  |  |  |  |
| Yes | 116 (6.3) | 206.6 (194.4-219.6) | 0.39 | 117 (6.4) | 20.2 (18.4-22.1) | 0.98 |
| No | 1713 (93.7) | 201.0 (197.9-204.3) |  | 1713 (93.6) | 20.2 (19.7-20.7) |  |
| **Trastuzumab therapy** |  |  |  |  |  |  |
| Yes | 53 (3.0) | 203.6 (186.1-222.6) | 0.77 | 54 (3.0) | 20.0 (17.5-22.9) | 0.94 |
| No | 1733 (97.0) | 200.8 (197.7-204.0) |  | 1733 (97.0) | 20.1 (19.7-20.6) |  |
| **Type of surgery** |  |  |  |  |  |  |
| Ablation | 721 (29.4) | 199.3 (194.3-204.3) | 0.07 | 722 (29.4) | 19.6 (18.8-20.3) | 0.24 |
| Breast-conserving therapy | 1734 (70.6) | 204.8 (201.5-208.2) |  | 1734 (70.6) | 20.1 (19.6-20.6) |  |
| **Body Mass Index (kg/m^2^)** |  |  |  |  |  |  |
| <25 | 1142 (46.6) | 200.8 (196.8-204.8) | 0.01 | 1140 (46.5) | 19.7 (19.2-20.3) | 0.56 |
| 25–<30 | 903 (36.8) | 201.9 (197.4-206.5) |  | 905 (36.9) | 20.0 (19.3-20.6) |  |
| ≥30 | 408 (16.6) | 212.7 (205.6-219.9) |  | 409 (16.7) | 20.4 (19.4-21.4) |  |
| **Menopausal status** |  |  |  |  |  |  |
| Surgical menopause | 230 (9.4) | 193.6 (184.1-203.6) | 0.05 | 231 (9.4) | 18.8 (17.4-20.2) | 0.10 |
| Natural menopause | 2226 (90.6) | 204.1 (201.1-207.1) |  | 2226 (90.6) | 20.0 (19.6-20.5) |  |
| **Current menopausal hormone therapy use** |  |  |  |  |  |  |
| Yes | 1107 (45.3) | 203.4 (199.3-207.6) | 0.79 | 1108 (45.3) | 20.3 (19.7-20.9) | 0.11 |
| No | 1337 (54.7) | 202.6 (198.9-206.5) |  | 1337 (54.7) | 19.6 (19.1-20.2) |  |
| **Education** |  |  |  |  |  |  |
| Low | 1458 (59.4) | 205.3 (201.6-209.0) | 0.17 | 1459 (59.3) | 20.0 (19.5-20.5) | 0.60 |
| Medium | 637 (25.9) | 200.9 (195.6-206.3) |  | 638 (26.0) | 20.0 (19.2-20.8) |  |
| High | 361 (14.7) | 198.6 (191.6-205.8) |  | 360 (14.7) | 19.4 (18.4-20.5) |  |
| **Alcohol intake** |  |  |  |  |  |  |
| Never | 559 (22.8) | 209.2 (203.3-215.2) | 0.01 | 559 (22.8) | 19.6 (18.8-20.4) | 0.66 |
| <19 g/day | 1536 (62.6) | 202.7 (199.3-206.3) |  | 1537 (62.6) | 20.0 (19.5-20.5) |  |
| ≥19 g/day | 359 (14.6) | 194.7 (187.9-201.8) |  | 359 (14.6) | 20.1 (19.1-21.2) |  |
| **Smoking status** |  |  |  |  |  |  |
| Never | 1329 (54.1) | 203.7 (199.9-207.6) | 0.78 | 1329 (54.1) | 20.2 (19.6-20.7) | 0.03 |
| Past | 668 (27.2) | 201.5 (196.3-206.8) |  | 668 (27.2) | 20.2 (19.5-21.0) |  |
| Current | 459 (18.7) | 203.8 (197.4-210.4) |  | 460 (18.7) | 18.8 (18.0-19.7) |  |
| **Cardiovascular disease** |  |  |  |  |  |  |
| Yes | 1211 (49.3) | 207.8 (203.8-212.0) | 0.0015 | 1210 (49.3) | 20.2 (19.6-20.7) | 0.27 |
| No | 1245 (50.7) | 198.6 (194.8-202.5) |  | 1247 (50.8) | 19.7 (19.1-20.3) |  |
| **Diabetes** |  |  |  |  |  |  |
| Yes | 220 (9.0) | 213.6 (204.0-223.6) | 0.02 | 220 (9.0) | 19.3 (18.1-20.7) | 0.34 |
| No | 2232 (91.0) | 202.0 (199.1-204.9) |  | 2233 (91.0) | 20.0 (19.6-20.4) |  |
| **Time between operation and blood draw** |  |  |  |  |  |  |
| The day of or before operation | 12 (0.5) | 183.0 (151.1-221.7) | <.0001 | 12 (0.5) | 16.0 (12.1-21.1) | <.0001 |
| 7 days | 333 (13.6) | 186.3 (179.6-193.2) |  | 332 (13.5) | 16.6 (15.8-17.6) |  |
| 7 days to 3 months | 816 (33.2) | 192.4 (187.8-197.1) |  | 817 (33.3) | 18.4 (17.8-19.1) |  |
| 3 to 6 months | 240 (9.8) | 224.5 (215.0-234.4) |  | 240 (9.8) | 21.3 (20.0-22.7) |  |
| 6 months to 1 year | 400 (16.3) | 221.1 (213.7-228.7) |  | 400 (16.3) | 21.3 (20.2-22.3) |  |
| 1 year or more | 655 (26.7) | 208.3 (202.8-214.0) |  | 656 (26.7) | 22.5 (21.6-23.4) |  |

^a^ Cross-sectional associations between clinical and lifestyle factors and OPG and TRAIL were evaluated in a linear regression model adjusted for age and center.

^b^Self-detected by palpation, secretion, and/or pain, routine examination includes mammography and ultrasound

Missing: mode of tumor detection 8; chemotherapy 26; radiotherapy 23; tamoxifen/ aomatase inhibitors therapy 99; bisphosphonate therapy 627; trastuzumab therapy 595; breast operation/ablation 1; current menopausal hormone therapy 12; body mass index 3; alcohol intake 2; diabetes 4.

Abbreviations: OPG, osteoprotegerin; n, number of observations; p, p-value/probability; TRAIL, TNF-related apoptosis-inducing ligand

**Table S2:** Hazard ratios and corresponding 95% confidence intervals for the association between circulating OPG concentration at blood collection and 5-years survival, stratified by ERPR status.

|  |  | **OPG** | | | |  | **ERPR-** | | | |  | **ER+PR- or ER-PR+** | | | |  | **ERPR+** | | | | |
| --- | --- | --- | --- | --- | --- | --- | --- | --- | --- | --- | --- | --- | --- | --- | --- | --- | --- | --- | --- | --- | --- |
|  |  | **n/events** | **HR^a^** | **(95% CI)** | **p** |  | **n/events** | **HR^b^** | **(95% CI)** | **p** |  | **n/events** | **HR^b^** | **(95% CI)** | **p** |  | **n/events** | **HR^b^** | **(95% CI)** | **p** | **Phet^c^** |
| **All-cause mortality** | | |  |  |  |  |  |  |  |  |  |  |  |  |  |  |  |  |  |  |  |
| Quartiles | 1 | 613/37 |  |  |  |  | 68/7 |  |  |  |  | 88/7 |  |  |  |  | 396/15 |  |  |  |  |
|  | 2 | 614/36 | 0.86 | (0.54-1.37) |  |  | 79/12 | 1.50 | (0.55-4.09) |  |  | 103/6 | 0.75 | (0.25-2.31) |  |  | 370/15 | 1.06 | (0.52-2.19) |  | 0.63 |
|  | 3 | 615/41 | 0.93 | (0.59-1.46) |  |  | 69/11 | 1.44 | (0.53-3.90) |  |  | 88/7 | 0.88 | (0.29-2.66) |  |  | 392/18 | 1.02 | (0.51-2.04) |  |  |
|  | 4 | 614/53 | 1.15 | (0.74-1.77) |  |  | 93/14 | 1.46 | (0.55-3.89) |  |  | 88/7 | 0.81 | (0.27-2.38) |  |  | 377/26 | 1.39 | (0.72-2.69) |  |  |
| Continuous^d^ | | 2456/167 | 1.17 | (0.85-1.59) | 0.34 |  | 309/44 | 1.55 | (0.79-3.06) | 0.20 |  | 367/27 | 1.11 | (0.50-2.48) | 0.79 |  | 1535/74 | 1.15 | (0.73-1.81) | 0.54 | 0.72 |
| **Breast cancer-specific mortality** | | | |  |  |  |  |  |  |  |  |  |  |  |  |  |  |  |  |  |  |
| Quartiles | 1 | 613/26 |  |  |  |  | 68/7 |  |  |  |  | 88/4 |  |  |  |  | 396/9 |  |  |  |  |
|  | 2 | 614/20 | 0.71 | (0.39-1.29) |  |  | 79/9 | 1.31 | (0.45-3.82) |  |  | 103/5 | 1.24 | (0.32-4.86) |  |  | 370/6 | 0.75 | (0.26-2.13) |  | 0.89 |
|  | 3 | 615/28 | 0.93 | (0.54-1.60) |  |  | 69/9 | 1.30 | (0.46-3.72) |  |  | 88/6 | 1.53 | (0.41-5.75) |  |  | 392/10 | 1.00 | (0.40-2.52) |  |  |
|  | 4 | 614/32 | 0.98 | (0.57-1.68) |  |  | 93/11 | 1.30 | (0.46-3.67) |  |  | 88/6 | 1.15 | (0.31-4.26) |  |  | 377/11 | 1.00 | (0.40-2.49) |  |  |
| Continuous^d^ | | 2456/106 | 1.08 | (0.73-1.60) | 0.71 |  | 309/36 | 1.25 | (0.59-2.62) | 0.56 |  | 367/21 | 1.50 | (0.62-3.62) | 0.37 |  | 1535/36 | 1.03 | (0.55-1.94) | 0.92 | 0.22 |
| **Recurrence-free survival^e^** | | |  |  |  |  |  |  |  |  |  |  |  |  |  |  |  |  |  |  |  |
| Quartiles | 1 | 558/48 |  |  |  |  | 62/6 |  |  |  |  | 83/9 |  |  |  |  | 376/30 |  |  |  |  |
|  | 2 | 566/47 | 0.93 | (0.62-1.39) |  |  | 75/15 | 2.14 | (0.79-5.75) |  |  | 99/9 | 0.87 | (0.34-2.21) |  |  | 353/21 | 0.73 | (0.42-1.29) |  | 0.48 |
|  | 3 | 555/56 | 1.15 | (0.77-1.70) |  |  | 63/15 | 2.65 | (0.98-7.14) |  |  | 81/9 | 1.07 | (0.41-2.78) |  |  | 371/30 | 0.93 | (0.55-1.55) |  |  |
|  | 4 | 560/64 | 1.17 | (0.79-1.73) |  |  | 88/20 | 2.51 | (0.96-6.57) |  |  | 84/10 | 1.05 | (0.42-2.66) |  |  | 352/33 | 1.06 | (0.63-1.77) |  |  |
| Continuous^d^ | | 2239/215 | 1.17 | (0.88-1.55) | 0.28 |  | 288/56 | 2.02 | (1.13-3.61) | 0.02 |  | 347/37 | 1.14 | (0.56-2.30) | 0.72 |  | 1452/114 | 1.02 | (0.70-1.48) | 0.94 | 0.10 |

Note: ERPR status was not available for 91 women who had neoadjuvant chemotherapy and 154 who were diagnosed tumor *in situ* or stage 0. ERPR-discordant includes ER+PR-, ER-PR+, ER+ or PR+ and data on other receptor unknown, or women treated with tamoxifen or aromatase inhibitor.

^a^Adjusted for age, nodal status, tumor size, and grade/ strata for center, ERPR status.

^b^Adjusted for age, nodal status, tumor size, and grade/ strata for center.

^c^Heterogeneity of estimates across ERPR status.

^d^Log-2 transformed.

^e^Patients without information on recurrence or with a tumor stages 3b, and 3c were excluded.

**Table S3:** Hazard ratios and corresponding 95% confidence intervals for the association between circulating TRAIL concentration at blood collection and 5-years survival, stratified by ERPR status.

|  |  | **TRAIL** | | | |  | **ERPR-** | | | |  | **ER+PR- or ER-PR+** | | | |  | **ERPR+** | | | | |
| --- | --- | --- | --- | --- | --- | --- | --- | --- | --- | --- | --- | --- | --- | --- | --- | --- | --- | --- | --- | --- | --- |
|  |  | **n/events** | **HR^a^** | **(95% CI)** | **p** |  | **n/events** | **HR^b^** | **(95% CI)** | **p** |  | **n/events** | **HR^b^** | **(95% CI)** | **p** |  | **n/events** | **HR^b^** | **(95% CI)** | **p** | **Phet^c^** |
| **All-cause mortality** | | |  |  |  |  |  |  |  |  |  |  |  |  |  |  |  |  |  |  |  |
| Quartiles | 1 | 614/53 |  |  |  |  | 66/12 |  |  |  |  | 99/6 |  |  |  |  | 387/24 |  |  |  |  |
|  | 2 | 614/34 | 0.63 | (0.41-0.97) |  |  | 78/11 | 0.78 | (0.34-1.80) |  |  | 93/6 | 1.15 | (0.36-3.70) |  |  | 385/13 | 0.55 | (0.28-1.09) |  | 0.53 |
|  | 3 | 615/40 | 0.76 | (0.50-1.16) |  |  | 81/13 | 1.07 | (0.47-2.46) |  |  | 83/8 | 1.80 | (0.61-5.35) |  |  | 393/17 | 0.64 | (0.34-1.19) |  |  |
|  | 4 | 614/40 | 0.76 | (0.50-1.16) |  |  | 84/8 | 0.61 | (0.25-1.51) |  |  | 92/7 | 1.80 | (0.61-5.35) |  |  | 371/20 | 0.87 | (0.48-1.59) |  |  |
| Continuous^d^ | | 2457/167 | 0.94 | (0.77-1.16) | 0.58 |  | 309/44 | 0.98 | (0.64-1.49) | 0.91 |  | 367/27 | 1.23 | (0.74-2.04) | 0.45 |  | 1536/74 | 0.91 | (0.67-1.25) | 0.57 | 0.20 |
| **Breast cancer-specific mortality** | | | |  |  |  |  |  |  |  |  |  |  |  |  |  |  |  |  |  |  |
| Quartiles | 1 | 614/27 |  |  |  |  | 66/7 |  |  |  |  | 99/6 |  |  |  |  | 387/9 |  |  |  |  |
|  | 2 | 614/19 | 0.71 | (0.39-1.28) |  |  | 78/10 | 1.21 | (0.45-3.24) |  |  | 93/4 | 0.78 | (0.21-2.95) |  |  | 385/3 | 0.37 | (0.10-1.37) |  | 0.51 |
|  | 3 | 615/31 | 1.26 | (0.74-2.13) |  |  | 81/12 | 1.91 | (0.71-5.15) |  |  | 83/6 | 1.30 | (0.40-4.23) |  |  | 393/11 | 1.14 | (0.47-2.77) |  |  |
|  | 4 | 614/29 | 1.14 | (0.67-1.95) |  |  | 84/7 | 0.99 | (0.34-2.87) |  |  | 92/5 | 0.97 | (0.29-3.31) |  |  | 371/13 | 1.59 | (0.67-3.77) |  |  |
| Continuous^d^ | | 2457/106 | 1.14 | (0.87-1.50) | 0.33 |  | 309/36 | 1.17 | (0.72-1.91) | 0.52 |  | 367/21 | 1.05 | (0.58-1.91) | 0.83 |  | 1536/36 | 1.15 | (0.72-1.83) | 0.56 | 0.68 |
| **Recurrence-free survival^e^** | | |  |  |  |  |  |  |  |  |  |  |  |  |  |  |  |  |  |  |  |
| Quartiles | 1 | 546/56 |  |  |  |  | 61/11 |  |  |  |  | 92/11 |  |  |  |  | 365/31 |  |  |  |  |
|  | 2 | 566/47 | 0.79 | (0.54-1.17) |  |  | 71/15 | 1.25 | (0.57-2.74) |  |  | 92/10 | 0.64 | (0.26-1.59) |  |  | 369/21 | 0.69 | (0.39-1.20) |  | 0.78 |
|  | 3 | 570/60 | 1.01 | (0.70-1.46) |  |  | 77/15 | 1.17 | (0.53-2.60) |  |  | 80/9 | 0.87 | (0.35-2.15) |  |  | 371/35 | 1.14 | (0.70-1.85) |  |  |
|  | 4 | 559/53 | 0.99 | (0.68-1.45) |  |  | 79/16 | 1.30 | (0.60-2.84) |  |  | 83/7 | 0.82 | (0.31-2.15) |  |  | 349/27 | 0.96 | (0.57-1.61) |  |  |
| Continuous^d^ | | 2241/216 | 1.06 | (0.88-1.28) | 0.55 |  | 288/57 | 1.23 | (0.84-1.81) | 0.28 |  | 347/37 | 0.90 | (0.57-1.42) | 0.82 |  | 1454/114 | 1.06 | (0.82-1.38) | 0.64 | 0.53 |

Note: ERPR status was not available for 91 women who had neoadjuvant chemotherapy and 154 who were diagnosed tumor *in situ* or stage 0. ERPR-discordant includes ER+PR-, ER-PR+, ER+ or PR+ and data on other receptor unknown, or women treated with tamoxifen or aromatase inhibitor.

^a^Adjusted for age, nodal status, tumor size, and grade/ strata for center, ERPR status.

^b^Adjusted for age, nodal status, tumor size, and grade/ strata for center.

^c^Heterogeneity of estimates across ERPR status.

^d^Log-2 transformed.

^e^Patients without information on recurrence or with a tumor stages 3b, and 3c were excluded.

**Table S4:** Sensitivity analysis for the association between circulating OPG and TRAIL concentration at blood collection and overall survival with exclusion of women diagnosed with *in situ* tumors, unknown stage, stage IIIb or higher breast cancer, and women treated with neoadjuvant chemotherapy, plus additional exclusions based on indicated time between surgery and blood collection.

|  |  | **Excluding women with blood drawn**  **within 7 days after breast surgery** | | | | | | | | |  | **Excluding women with blood drawn**  **within 3 months after breast surgery** | | | | | | | | |
| --- | --- | --- | --- | --- | --- | --- | --- | --- | --- | --- | --- | --- | --- | --- | --- | --- | --- | --- | --- | --- |
|  |  | **OPG** | | | |  | **TRAIL** | | | |  | **OPG** | | | |  | **TRAIL** | | | |
|  |  | **n/events** | **HR^a^** | **(95% CI)** | **p** |  | **n/events** | **HR^a^** | **(95% CI)** | **p** |  | **n/events** | **HR^a^** | **(95% CI)** | **p** |  | **n/events** | **HR^a^** | **(95% CI)** | **p** |
| **All-cause mortality** | | |  |  |  |  |  |  |  |  |  |  |  |  |  |  |  |  |  |  |
| Quartiles | 1 | 423/67 |  |  |  |  | 421/84 |  |  |  |  | 202/25 |  |  |  |  | 216/37 |  |  |  |
|  | 2 | 450/73 | 0.97 | (0.70-1.36) |  |  | 449/75 | 0.80 | (0.58-1.09) |  |  | 270/48 | 1.42 | (0.87-2.32) |  |  | 256/47 | 0.99 | (0.64-1.53) |  |
|  | 3 | 445/79 | 1.03 | (0.74-1.44) |  |  | 461/93 | 0.99 | (0.73-1.33) |  |  | 279/44 | 1.26 | (0.77-2.08) |  |  | 285/52 | 1.01 | (0.66-1.55) |  |
|  | 4 | 479/112 | 1.27 | (0.92-1.74) |  |  | 468/80 | 0.85 | (0.63-1.16) |  |  | 336/72 | 1.56 | (0.97-2.50) |  |  | 331/53 | 0.93 | (0.61-1.41) |  |
| Continuous^b^ | | 1797/331 | 1.30 | (1.03-1.63) | 0.03 |  | 1799/332 | 0.97 | (0.83-1.13) | 0.67 |  | 1087/189 | 1.43 | (1.05-1.94) | 0.02 |  | 1088/189 | 0.95 | (0.77-1.16) | 0.62 |
| **Breast cancer-specific mortality** | | | | |  |  |  |  |  |  |  |  |  |  |  |  |  |  |  |  |
| Quartiles | 1 | 423/44 |  |  |  |  | 421/40 |  |  |  |  | 202/15 |  |  |  |  | 216/15 |  |  |  |
|  | 2 | 450/36 | 0.77 | (0.49-1.19) |  |  | 449/36 | 0.83 | (0.53-1.30) |  |  | 270/22 | 1.12 | (0.58-2.19) |  |  | 256/18 | 0.92 | (0.46-1.84) |  |
|  | 3 | 445/41 | 0.87 | (0.56-1.35) |  |  | 461/59 | 1.38 | (0.92-2.06) |  |  | 279/22 | 1.14 | (0.58-2.24) |  |  | 285/36 | 1.68 | (0.91-3.09) |  |
|  | 4 | 479/54 | 1.01 | (0.66-1.53) |  |  | 468/41 | 0.98 | (0.63-1.53) |  |  | 336/38 | 1.51 | (0.81-2.81) |  |  | 331/28 | 1.21 | (0.64-2.28) |  |
| Continuous^b^ | | 1797/175 | 1.10 | (0.80-1.50) | 0.57 |  | 1799/176 | 1.09 | (0.88-1.35) | 0.43 |  | 1087/97 | 1.35 | (0.87-2.09) | 0.18 |  | 1088/97 | 1.11 | (0.82-1.48) | 0.51 |
| **Recurrence-free survival** | | |  |  |  |  |  |  |  |  |  |  |  |  |  |  |  |  |  |  |
| Quartiles | 1 | 418/92 |  |  |  |  | 416/106 |  |  |  |  | 198/35 |  |  |  |  | 213/45 |  |  |  |
|  | 2 | 447/98 | 0.96 | (0.72-1.28) |  |  | 448/99 | 0.84 | (0.64-1.10) |  |  | 268/63 | 1.33 | (0.87-2.01) |  |  | 256/55 | 0.93 | (0.62-1.38) |  |
|  | 3 | 444/107 | 1.07 | (0.81-1.43) |  |  | 456/116 | 1.01 | (0.78-1.32) |  |  | 278/67 | 1.46 | (0.96-2.22) |  |  | 281/72 | 1.20 | (0.83-1.75) |  |
|  | 4 | 475/128 | 1.14 | (0.86-1.51) |  |  | 466/105 | 0.90 | (0.68-1.17) |  |  | 334/80 | 1.29 | (0.85-1.94) |  |  | 329/73 | 1.06 | (0.73-1.54) |  |
| Continuous^b^ | | 1784/425 | 1.19 | (0.97-1.45) | 0.09 |  | 1786/426 | 1.01 | (0.88-1.15) | 0.92 |  | 1078/245 | 1.28 | (0.98-1.68) | 0.07 |  | 1079/245 | 1.06 | (0.88-1.27) | 0.55 |

^a^Adjusted for age, nodal status, tumor size, and grade/ strata for center and ERPR status

^b^Log-2 transformed

**Table S5:** Sensitivity analysis for the association between circulating OPG and TRAIL concentration at blood collection and 5-years survival with exclusion of women diagnosed with *in situ* tumors, unknown stage, stage IIIb or higher breast cancer, and women treated with neoadjuvant chemotherapy, plus additional exclusions based on indicated time between surgery and blood collection.

|  |  | **Excluding women with blood drawn**  **within 7 days after breast surgery** | | | | | | | | |  | **Excluding women with blood drawn**  **within 3 months after breast surgery** | | | | | | | | | |
| --- | --- | --- | --- | --- | --- | --- | --- | --- | --- | --- | --- | --- | --- | --- | --- | --- | --- | --- | --- | --- | --- |
|  |  | **OPG** | | | |  | **TRAIL** | | | |  | **OPG** | | | |  | **TRAIL** | | | | |
|  |  | **n/events** | **HR^a^** | **(95% CI)** | **p** |  | **n/events** | **HR^a^** | **(95% CI)** | **p** |  | **n/events** | **HR^a^** | **(95% CI)** | **p** |  | **n/events** | **HR^a^** | **(95% CI)** | **p** |  |
| **All-cause mortality** | | |  |  |  |  |  |  |  |  |  |  |  |  |  |  |  |  |  |  |  |
| Quartiles | 1 | 423/17 |  |  |  |  | 421/26 |  |  |  |  | 202/4 |  |  |  |  | 216/7 |  |  |  |  |
|  | 2 | 450/25 | 1.22 | (0.65-2.27) |  |  | 449/18 | 0.64 | (0.35-1.18) |  |  | 270/17 | 2.96 | (0.99-8.89) |  |  | 256/8 | 0.90 | (0.32-2.49) |  |  |
|  | 3 | 445/22 | 1.04 | (0.55-1.98) |  |  | 461/26 | 0.88 | (0.51-1.51) |  |  | 279/12 | 1.94 | (0.61-6.11) |  |  | 285/17 | 1.54 | (0.64-3.76) |  |  |
|  | 4 | 479/34 | 1.28 | (0.70-2.35) |  |  | 468/28 | 1.03 | (0.60-1.76) |  |  | 336/16 | 1.77 | (0.58-5.42) |  |  | 331/17 | 1.53 | (0.63-3.72) |  |  |
| Continuous^b^ | | 1797/98 | 1.36 | (0.89-2.07) | 0.15 |  | 1799/98 | 1.09 | (0.82-1.44) | 0.56 |  | 1087/49 | 1.3 | (0.70-2.44) | 0.41 |  | 1088/49 | 1.38 | (0.90-2.12) | 0.14 |  |
| **Breast cancer-specific mortality** | | | | |  |  |  |  |  |  |  |  |  |  |  |  |  |  |  |  |  |
| Quartiles | 1 | 423/12 |  |  |  |  | 421/13 |  |  |  |  | 202/2 |  |  |  |  | 216/3 |  |  |  |  |
|  | 2 | 450/14 | 0.99 | (0.45-2.17) |  |  | 449/8 | 0.58 | (0.24-1.41) |  |  | 270/9 | 3.24 | (0.68-15.47) |  |  | 256/1 | 0.23 | (0.02-2.24) |  |  |
|  | 3 | 445/16 | 1.15 | (0.53-2.48) |  |  | 461/19 | 1.33 | (0.65-2.71) |  |  | 279/8 | 3.09 | (0.63-15.17) |  |  | 285/13 | 2.34 | (0.66-8.32) |  |  |
|  | 4 | 479/17 | 0.87 | (0.40-1.88) |  |  | 468/19 | 1.45 | (0.71-2.95) |  |  | 336/10 | 2.31 | (0.48-11.07) |  |  | 331/12 | 2.15 | (0.60-7.77) |  |  |
| Continuous^b^ | | 1797/59 | 1.07 | (0.62-1.86) | 0.81 |  | 1799/59 | 1.32 | (0.90-1.93) | 0.16 |  | 1087/29 | 1.30 | (0.55-3.08) | 0.55 |  | 1088/29 | 1.93 | (1.04-3.56) | 0.04 |  |
| **Recurrence-free survival** | | |  |  |  |  |  |  |  |  |  |  |  |  |  |  |  |  |  |  |  |
| Quartiles | 1 | 418/37 |  |  |  |  | 416/40 |  |  |  |  | 198/10 |  |  |  |  | 213/12 |  |  |  |  |
|  | 2 | 447/40 | 0.98 | (0.62-1.53) |  |  | 448/40 | 0.94 | (0.61-1.46) |  |  | 268/21 | 1.52 | (0.71-3.25) |  |  | 256/13 | 0.81 | (0.37-1.79) |  |  |
|  | 3 | 444/42 | 1.02 | (0.65-1.61) |  |  | 456/51 | 1.16 | (0.77-1.76) |  |  | 278/25 | 1.85 | (0.87-3.91) |  |  | 281/30 | 1.57 | (0.80-3.08) |  |  |
|  | 4 | 475/58 | 1.21 | (0.79-1.87) |  |  | 466/47 | 1.15 | (0.75-1.75) |  |  | 334/30 | 1.48 | (0.71-3.09) |  |  | 329/31 | 1.63 | (0.83-3.19) |  |  |
| Continuous^b^ | | 1784/177 | 1.24 | (0.91-1.71) | 0.18 |  | 1786/178 | 1.16 | (0.94-1.44) | 0.18 |  | 1078/86 | 1.37 | (0.85-2.20) | 0.19 |  | 1079/86 | 1.39 | (1.00-1.93) | 0.05 |  |

^a^Adjusted for age, nodal status, tumor size, and grade/ strata for center and ERPR status

^b^Log-2 transformed

**Figure S1**: OPG and TRAIL concentrations by time between breast cancer operation and blood collection.

| 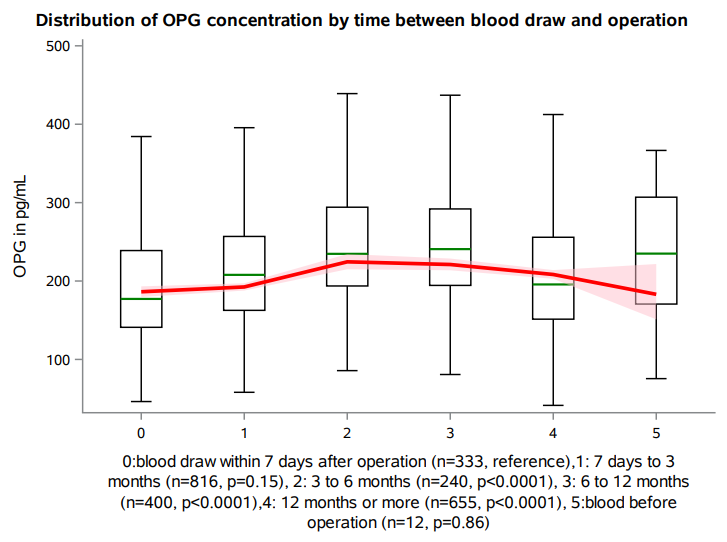 | 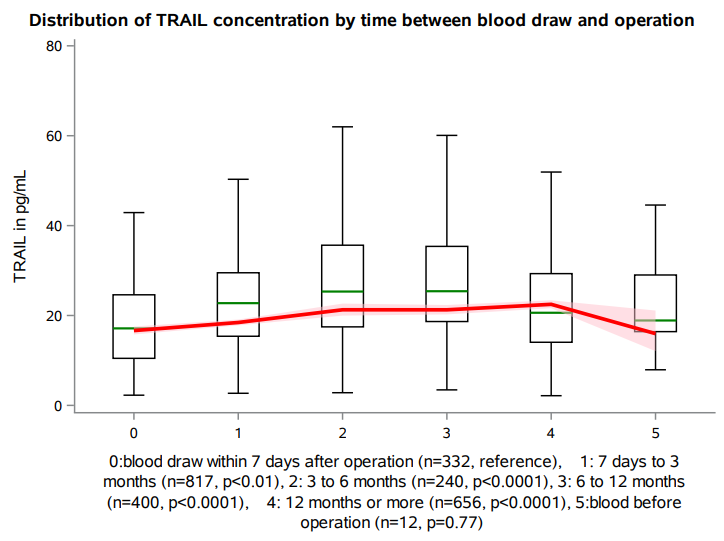 |
| --- | --- |

Legend: OPG and TRAIL concentrations represented by boxplots, and geometric means represented by the red line.
